# An assessment of the Dutch experience with health insurers acting as healthcare advisors

**DOI:** 10.1371/journal.pone.0224829

**Published:** 2019-11-08

**Authors:** A. Victoor, A. E. M. Brabers, T. E. M. van Esch, J. D. de Jong

**Affiliations:** 1 Nivel (Netherlands Institute for Health Services Research), Utrecht, the Netherlands; 2 Maastricht University, Maastricht, the Netherlands; University of Malta Faculty of Health Sciences, MALTA

## Abstract

**Introduction:**

With managed competition, selective contracting and the appointing of preferred providers are important instruments for health insurers to improve their bargaining position in the healthcare purchasing market. Insurers can offer enrollees extra services, such as advice about their healthcare, to attract them, ensure that they remain loyal, and to channel them to preferred providers. We investigate which advice services insurers in the Dutch system of managed competition offer enrollees, how they inform them about services, and if enrollees use and appreciate them.

**Materials and methods:**

From November to December 2017, two researchers independently analyzed the websites of all health insurers in the Netherlands. We also conducted a questionnaire study among 1,500 members (response 44.5%, N = 668) of the Nivel Dutch Health Care Consumer Panel.

**Results and discussion:**

All insurers offer one or more services. Most enrollees do not know if their insurer offers advice (67–87% per service). Twelve per cent (N = 76) of the enrollees indicate that they ever made use of a service, mostly regarding the choice of provider (N = 42). Respondents who used healthcare advice were satisfied with it. Of all enrollees, 41% indicate that they would probably/certainly, contact their insurer for advice and 37% would appreciate it if their insurer approached them. Among enrollees, 40% indicated the potential advice has some or a major influence on their choice of insurer.

**Conclusions:**

While all insurers offer at least one service, enrollees generally are unaware of them. Only a minority ever made use of such a service. However, a reasonable proportion do appreciate their insurers’ advice services and indicate that they would like to have contact with their insurer if they need care. Insurers do not appear to make the best use of the potential for giving healthcare advice and need to think about ways to increase coverage of those services.

## Introduction

### Background

Market incentives have been introduced into healthcare in many countries in order to engineer a shift from a supply-oriented to a demand-oriented healthcare system. This shift is aimed at restraining healthcare costs and improving the quality of care. Several countries used the concept of managed competition as a framework for the reform, such as the USA, the Netherlands, and Germany. Managed competition is a strategy for purchasing healthcare in order to obtain maximum value for price, for the purchasers (insurers) and consumers (enrollees) [1, 2].

Health insurers have a crucial role to play in managed competition. In theory, they prudently purchase care on behalf of their enrollees based on both the quality and costs of care. They compete for enrollees on the health insurance market by providing them with the best offer, i.e. good quality of both care and service and a good price. To realize this, insurers are allowed to negotiate with providers on price and quality of care on the healthcare purchasing market. Insurers have a stronger bargaining position in negotiation with care providers if they are able to channel their enrollees towards specific care providers, in exchange for favorable contractual conditions with these providers. These conditions may include a competitive price and better or more efficient care [3–7].

Insurers could use different instruments in order to channel enrollees towards specific care providers [8–10]. One of the instruments is selective contracting. This means that they only conclude a contract with a subset of providers based on the costs and quality of care. They do not have to reimburse, fully, the costs of care incurred at non-contracted providers. However, it is argued that this impairs patients’ freedom to choose a provider. Research has been undertaken on the effects of selective contracting [9, 11]. Both provider and consumer organizations criticize restrictions on the freedom of provider choice [12].

Another instrument is appointing preferred providers. This means that patients retain their freedom to choose a provider, but insurers stimulate enrollees to opt for providers with whom they have made agreements on price, quality and/or volume of care [6, 8–10]. We focus on this instrument in the current study.

Insurers can stimulate enrollees to opt for specific providers in different ways. Examples may include giving them an incentive based on the quality of their care, such as giving ‘good’ providers a quality mark. Another example is offering positive financial incentives. Such incentives may include allowing enrollees, who opt for a specific provider, exemption from paying a deductible, or giving a discount on co-payments [8, 9, 13]. Another method is giving enrollees advice, without obligation, about which provider they consider to be best suited to the needs and wishes of the individual patient [14]. We currently know little about this method and insurers currently make little use of it. They assume that enrollees do not trust them to act in their best interest and will therefore not follow-up their advice [14, 15]. For many patients their general practitioner (GP), is the most important source of healthcare information [16, 17]. But since GPs may not discuss referral options adequately with their patients, they should not be patients’ only available source of information. Furthermore, giving enrollees advice, appears to be a very effective method of channeling for insurers [14]. At the same time, such healthcare advice services could enhance their appeal to people and attract those who intend to switch insurers or foster the commitment of their enrollees [18–20]. This improves their bargaining position towards other insurers. Such healthcare advice services that Dutch insurers offer their enrollees may include advice about a suitable provider or waiting list mediation [21].

### Research focus

In order to have a strong bargaining position vis-à-vis providers, it is important that insurers are able to channel enrollees towards contracted care providers or providers with whom they have made certain agreements. Giving advice is one method of channeling. At the same time, offering different advice services improves their bargaining position towards other insurers. The reason for this is that it enhances their appeal to enrollees and foster their commitment. Therefore, we should gain insight into what insurers currently do to advise their enrollees about matters involving their healthcare and, if enrollees appreciate these efforts. In the current paper, we provide insight into which healthcare advice services insurers offer their enrollees, how they inform enrollees about the availability of those services, and whether enrollees use and appreciate the services. Through focusing on the Netherlands (see Table 1 for more information about the Dutch healthcare system), we address the following research questions:

1. How, and to what degree, do insurers offer enrollees healthcare advice?
2. How, and to what degree, do insurers inform enrollees about the healthcare advice services they offer?
3. Do enrollees use and appreciate insurers’ healthcare advice services?

**Table 1:** Relevant aspects of the Dutch healthcare system with managed competition.

Relevant aspects of the Dutch healthcare system are:
- Every Dutch citizen is required to have a basic health plan (compulsory health insurance). The contents of the basic benefits package are determined by the government with regards to care, not in terms of providers.
- Health insurers have to accept all applicants. However, they are compensated financially for elderly people and those at high risk of disease, through a system of risk equalization.
- Citizens can buy supplementary insurance which can reimburse the costs of additional healthcare and co-payments.
- Enrollees are allowed to switch health plans every year.
- Enrollees have a mandatory deductible of €385 per person per year in 2016–2019 and a voluntary deductible up to €500 per person per year.
- Patients share some of the costs of selected services, such as medical devices, via co-payments.
- Insurers are allowed to contract care providers selectively and do not have to reimburse fully those who are not contracted. The norm is a minimum coverage for non-contracted care providers of 75%-80% of the mean tariff for that care at contracted providers.

### Scientific and social relevance

There may be research about the advisory role healthcare providers play, but few papers exist about organizations such as health insurers (e.g. [14]), or other parties, including employers, who may act as healthcare advisors (e.g. [22]). However, these parties seem to be able to influence patients’ provider choices significantly. We are unaware of any papers that investigate the different ways in which insurers act as healthcare advisors, from both the insurers’ and the patients’ perspective.

In the Netherlands, managed competition was introduced back in 2006. Since that time, insurers have been developing healthcare services to offer enrollees in order to attract and retain them. This in turn would strengthen their bargaining position in both the healthcare purchasing and health insurance markets. As a result of this, competition in these markets should, in theory, contribute to an improvement of the quality and efficiency of healthcare. For instance, by resulting in shorter waiting lists. Other countries may also have introduced waiting times norms, but these countries either do not allow patients to choose an alternative provider or the patients have to search for another provider themselves [23]. By contrast, Dutch insurers help their enrollees to find a suitable and accessible provider. Furthermore, the Netherlands is unique in that it combines elements of various healthcare systems from, for instance the Nordic countries, the USA, and the UK. This enables insurers to aim to be the prudent buyers of care on behalf of their enrollees, and patients to have a free choice of insurer, provider, and treatment. For these reasons, investigating the degree to which health insurers act as healthcare advisors in the Netherlands, and how patients feel about this, adds to the existing literature and has a relevance beyond the borders of the Netherlands.

Depending on the results, insurers and other parties who act as patients’ healthcare advisors in a system of managed competition could use these insights as a basis for adjusting the way in which they offer advice to their enrollees in order to align it to enrollees’ preferences. For instance, if we find that enrollees do appreciate their insurers’ healthcare advice services and often see the services on their insurer’s website, this might also apply to other countries. Insurers, globally, could offer extra services to build loyalty with enrollees and mention the services on their website. In addition to the advantages for enrollees, it might also increase insurers’ attractiveness to current or potential enrollees. This strengthens insurers’ position on the health insurance market. Additionally, healthcare advice might enable insurers to channel patients to contracted or preferred providers. This strengthens their bargaining position and, ultimately, might improve healthcare quality and efficiency through competition in the market for purchasing healthcare.

## Materials and methods

To answer our research questions, we used a combination of methods. We analyzed the websites of all health insurers in the Netherlands and conducted a questionnaire study amongst members of Nivel Dutch Health Care Consumer Panel [24]. This panel provides information on opinions and knowledge about healthcare, and expectations and experiences with healthcare. At the time of this study, the Consumer Panel consisted of approximately 12,000 people aged 18 years and older. The background characteristics for all panel members, such as gender, age and the highest level of education completed, are assessed as members join the panel. Each year, approximately eight to ten investigations are conducted. Each individual panel member receives a questionnaire approximately three times a year and can resign from the panel at any time. There is no possibility of people signing up for the panel on their own initiative. The Dutch Health Care Consumer Panel is renewed on a regular basis. More details on the recruitment and selection of panel members are reported elsewhere [24]. Data are analyzed anonymously, and processed according to the privacy policy of the Dutch Healthcare Consumer Panel, which complies with the General Data Protection Regulation (GDPR). According to Dutch legislation, there is no legal requirement either to obtain informed consent, nor gain approval by a medical ethics committee, for conducting research through the panel [25].

### Website analysis

We analyzed all health insurers’ websites for enrollees which were operating in the Dutch health insurance market at a time in which enrollees could switch insurers during 2017 to 2018 (32 entities in total, S1 Table). The analysis of the websites consisted of several steps:

1. Two researchers independently analyzed all websites in November to December 2017 using an Excel template to record the data in a uniform manner. The websites were searched for several topics, of which healthcare advice services was one. With regard to this topic the following questions were scored by the researchers for each of the four forms of healthcare advice of Table 2 which were examined:
  - Does the insurer offer the healthcare advice service? (yes, no, unknown (meaning ‘no information available on the website’));
  - How does the insurer offer enrollees healthcare advice? (multiple choice: the service is available on the website, per telephone, per mail, na, in another way);
  - When does the insurer offer enrollees advice? (multiple choice: the service is always available on the website, if enrollees request the service, na, other).
2. The researchers compared their results and discussed differences in order to reach a consensus, per website, at the end of December 2017.
3. Insurers were asked to assess if the information was complete and to suggest adjustments if necessary. Fifteen insurers suggested adjustments and we adjusted the data for eleven insurers. In the case of the other four insurers, we only made clarifications to avoid misunderstandings.

**Table 2:** The definitions of the healthcare advice services which we used in our study.

Healthcare advice service	Definition
Advice about the most suitable provider	Advice, without obligation, about which provider the insurer considers to be best suited to the enrollee’s needs and wishes. To meet this definition, the advice service should go beyond an automatic search function on the insurer’s website as here a provider’s suitability is based solely on its proximity and if it is contracted by the insurer.
Waiting list mediation	Waiting lists are longer with certain providers than they are with others. In the event of a long waiting list for treatment with the enrollee’s care provider, they can contact their insurer to apply for waiting time mediation. The insurer will then assess whether an alternative provider is capable of providing appropriate treatment. The mediation service contacts all the available alternatives to investigate whether the enrollee can be treated earlier. Enrollees are free to choose whether or not they wish to opt for one or other of the alternatives.
Assistance with arranging care	Assistance with arranging care, for instance with arranging a second opinion, district nursing, maternity care or informal care.
Assistance with preparing a consultation with a physician	Assistance with preparing a consultation with a physician, for instance discussing with the enrollee the questions they could ask their physician during the consultation.

### Questionnaire study

In November 2017, we sent a questionnaire to 1,500 members of the Nivel Dutch Health Care Consumer Panel (S1 File). The questionnaire encompassed multiple themes, including the focus of this study, healthcare advice by the health insurer. The sample was representative of the general Dutch population aged 18 years and older, regarding age and gender. Depending on their preference, members received the questionnaire online (n = 756) or on paper (n = 744). Panel members were free to answer the questions or not. Non-responding members received one (on paper) or two (online) reminders. The closing date was four weeks after the initial communication. The response was 44.5% (N = 668).

### Statistical analysis

We analyzed the data, for both the websites and the questionnaire study, with Stata version 15. With regard to the survey data, mainly descriptive statistics were performed. Relevant differences between groups, regarding sex, age, education level and subjective health, were tested with Chi-squared tests with a significance level of 0.05. We applied a weighting factor for age and gender, with a range of 0.74–1.63, as our respondents to the questionnaire study were slightly older than the general Dutch population.

## Results and discussion

### Results

Table 3 describes the demographic characteristics of the participants. The majority was male, between 40 and 65 years old, had a medium education and a good subjective health.

**Table 3:** Background characteristics of the respondents (n (%)).

**Sex**	
Man	344(51.5%)
**Age in years**	
18–39	133(19.9%)
40–64	363(54.3%)
65 or older	172(25.8%)
**Educational level**	
Low^1^	93(14.4%)
Medium^2^	317(49.0%)
High^3^	237(36.6%)
**Subjective health**	
Excellent	38(6.2%)
Very good	126(20.5%)
Good	343(55.9%)
Fair	93(15.2%)
Poor	14(2.3%)

^1^Low = none, primary school or pre-vocational training

^2^Medium = secondary or vocational education

^3^High = professional higher education or university.

#### Research question 1: How, and to what degree do insurers offer enrollees healthcare advice?

**Fig 1:**
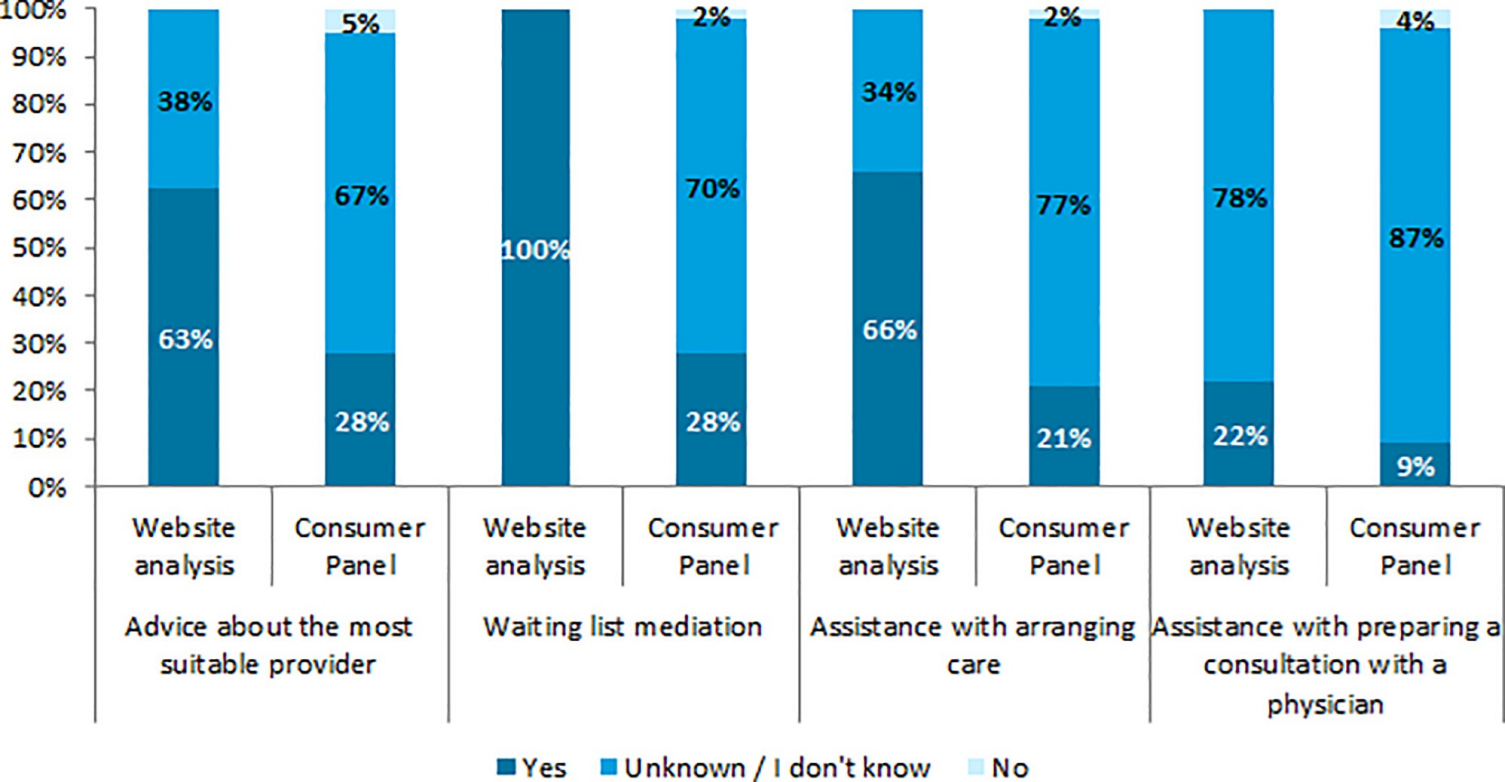
The percentage of insurers who offer each healthcare advice service, according to the website analysis (N = 32) and according to the questionnaire (N = 614–622, weighted).

The website analysis indicates that all insurers offer one or more healthcare advice services (Fig 1). Waiting list mediation is offered by all (n = 32 (100%)), assistance with preparing a consultation with a physician is offered least often (n = 7 (22%)). For about two-thirds (65%) of the insurers who offer healthcare advice, enrollees could request healthcare advice in different ways (phone, online form, email, website, social media, private online portal). This advice was offered most often by telephone (88%) and least often in person at the insurer’s office (1%) (data not shown in Fig 1). No differences were found between respondents regarding sex, age, education level and subjective health.

#### Research question 2: How, and to what degree do insurers inform enrollees about the healthcare advice services they offer?

Although all insurers offer one or more healthcare advice services, the results from the questionnaire indicate that most enrollees do not know if their insurer offers healthcare advice (67–87% per service, Fig 1). Enrollees who do know if their insurer offers a form of healthcare advice, most often saw it on their insurer’s website (5–18% per service). Enrollees aged 65 years or older more often (15%) indicate that they can ask their insurer to help them prepare a consultation with their physician than enrollees aged 18–39 (4%) or 50–64 (10%) years old. No differences were found between respondents regarding sex, education level and subjective health.

#### Research question 3: Do enrollees use and appreciate insurers’ healthcare advice services?

Twelve per cent (N = 76) of the enrollees indicate that they made use of a healthcare advice service from their insurer at least once (Table 4). This is most often advice about a suitable healthcare provider (N = 42). And least often, assistance with preparing a consultation with a physician (N = 7). Respondents who used healthcare advice were generally satisfied or very satisfied with the service (range 75%(N = 3)-81%(N = 26) for the different services, data not shown in Table 4).

**Table 4:** Would you indicate, per healthcare advice service, if you used it at least once (N = 623, weighted)?

Healthcare service	n
Respondent received at least one of the services below	76 (12%)*
Advice about the most suitable provider	42
Waiting list mediation	25
Assistance with arranging care	19
Assistance with preparing a consultation with a physician	7

*The total number adds up to 93 because respondents could select more than one option.

**Fig 2:**
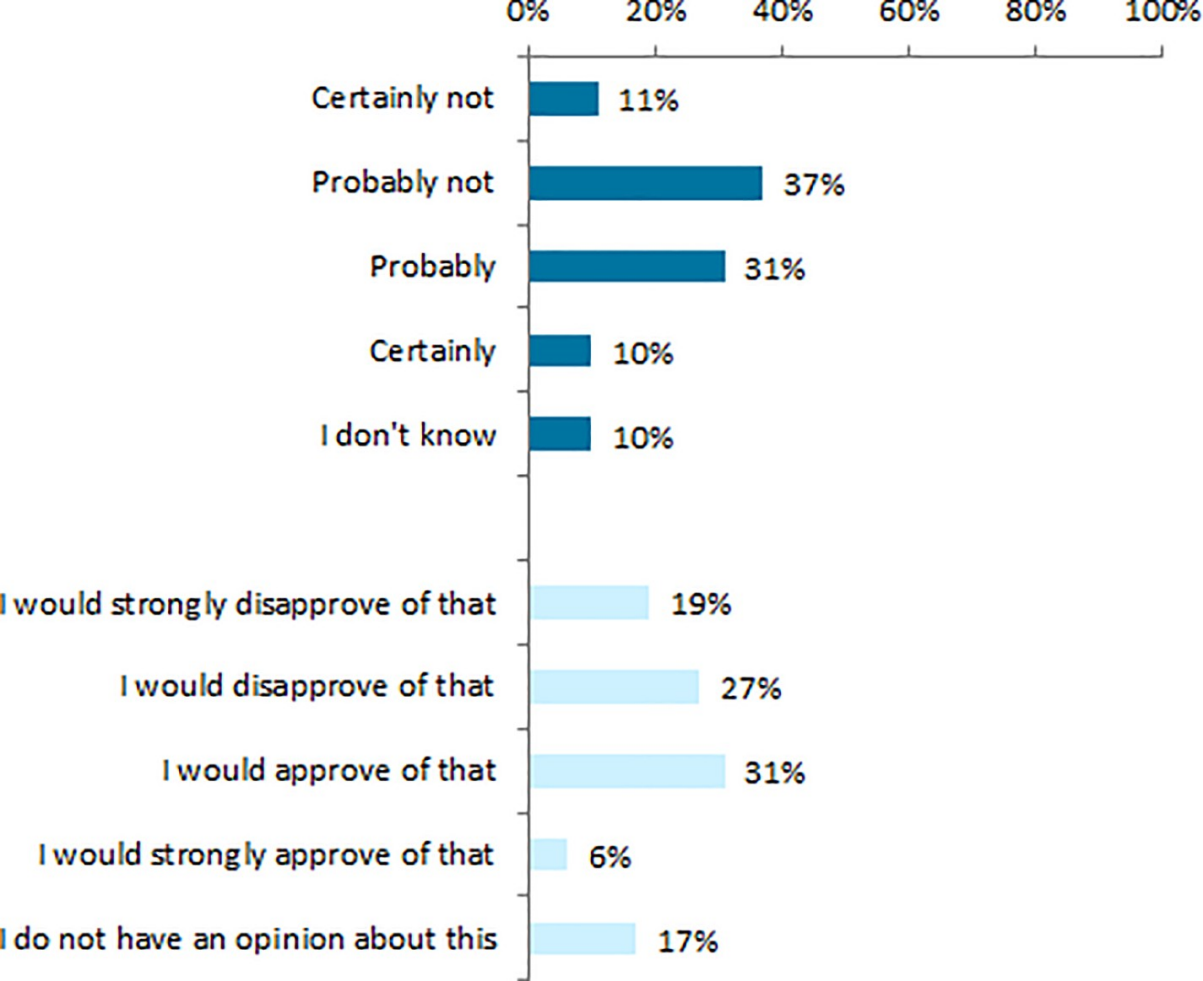
Would you contact your insurer if you needed one of the healthcare advice services? (N = 625, weighted) (dark blue). And would you appreciate it if your health insurer would approach you with healthcare advice, for instance about the quality of a specific healthcare provider? (N = 625, weighted) (light blue).

Forty-one per cent of all enrollees indicated that they would probably, or certainly, contact their insurer for healthcare advice if they needed it (Fig 2). Almost half (48%) of the enrollees indicated that they would probably, or certainly, not approach their insurer if they needed advice. Higher educated enrollees (58%) more often indicate that they would probably, or certainly, *not* approach their insurer than middle (41%) and low educated (38%) enrollees. No differences were found between respondents regarding sex, age and subjective health. Of the group who would probably, or certainly, contact their insurer, 37% did not know that their insurer offers any healthcare advice service. Of the enrollees, who would probably or certainly *not* approach their insurer, 53% did not know about these services. More than one-third (37%) would appreciate it if their insurer approached them with healthcare advice (Fig 2). Men appreciated this more often than women (46% versus 31%). No differences were found between respondents regarding age, education level and subjective health.

**Fig 3:**
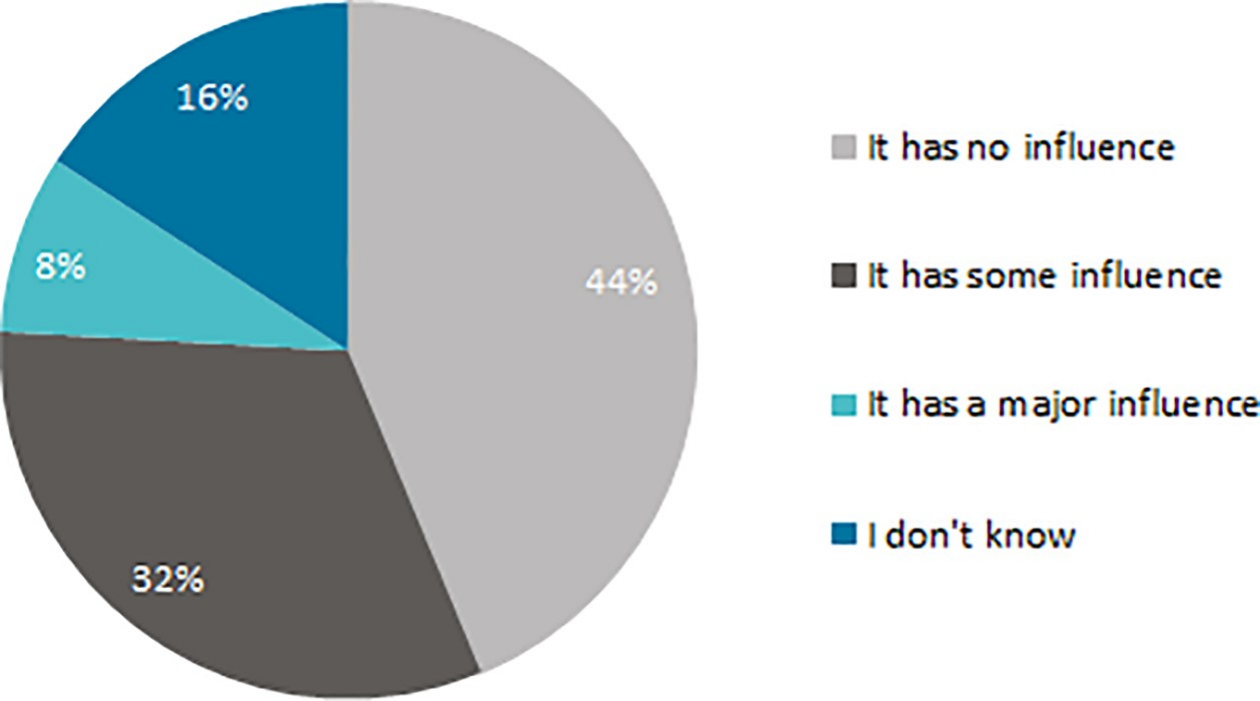
To what degree does the potential that your insurer could offer healthcare advice influence your choice of insurer? (N = 625, weighted).

The potential healthcare advice of their insurer does not have a significant influence upon enrollees’ insurer choice: 44% indicated no influence at all, and 32% indicated some influence (Fig 3). Highly educated enrollees (51%) indicated more often than middle (42%) and low educated (34%) enrollees that the potential advice did not influence their choice of insurer. Of the group who indicate that the potential advice would slightly, or greatly influence their insurer choice, more than one-third (37% and 40%) did not know whether their insurer offers healthcare advice. No differences were found between respondents regarding sex, age and subjective health.

## Discussion

While all health insurers offer one or more healthcare advice services, in general enrollees did not know if their health insurer offers such a service. In addition, only a minority indicated that they ever made use of any healthcare advice service from their insurer. Yet insurers are now expected to play an important role: that of being prudent buyers of healthcare on behalf of their enrollees. To be able to meet this expectation, it is essential for them to improve their bargaining position in the healthcare purchasing market, both by selective contracting and by appointing preferred providers [8, 13]. In order for these instruments to work, insurers need to channel enrollees to contracted or preferred providers. For instance by offering them healthcare advice about the most suitable provider. Offering enrollees this kind of extra services could, at the same time, improve their position on the healthcare insurance market. This is because it helps them to attract enrollees and keep them satisfied.

Our results indicate that the healthcare advice of the insurer might influence enrollees’ provider choice at least to some degree. So, offering enrollees healthcare advice seems to be a real opportunity to channel enrollees. A large group of the enrollees questioned indicated that they would probably, or certainly, contact their insurer for healthcare advice if they needed advice, or would appreciate it if their insurer approached them with healthcare advice. It might not be very surprising that few enrollees currently used a healthcare advice service from their insurer since it is only relevant for those who have to opt for a provider. One study indicated that in the Netherlands, per 1,000 patients registered with a GP, only 303 were referred to a medical specialist [26]. Respondents who did use healthcare advice are generally satisfied with the service. Additionally, a large group of enrollees (40%) indicated that the potential healthcare advice of their insurer influences their insurer choice. Therefore, offering enrollees healthcare advice services also seems to help ensure that enrollees remain loyal and to help attract new enrollees.

We also found that all healthcare insurers offer one or more healthcare advice services. Most often waiting list mediation and assistance with arranging care are offered. Enrollees can often request healthcare advice in different ways and those who knew about the services saw the possibility on their insurer’s website. Thus, insurers are certainly making efforts to meet everyone’s expectations. However, since few enrollees know about the existence of such services, insurers have so far not made full use of the potential for giving their enrollees healthcare advice. Therefore, insurers’ websites only seem to reach a limited proportion of the population. Other channels need to be explored to increase coverage, such as flyers or actively approaching enrollees.

However, although insurers may wish to increase coverage, they certainly want to avoid irritating enrollees. Not everyone will appreciate the insurers’ healthcare advice, since almost half of the enrollees indicated that they do not appreciate it if their insurer approaches them with advice, or would not approach their insurer to request advice. This is probably related to the fact that many enrollees generally distrust health insurers, do not trust their insurer to act in their best interest, nor to buy care on their behalf [15, 27]. It is known that patients often rely on their GP for matters involving their health [16, 17]. Other research, however, also found that a large group of enrollees appreciated and followed up advice from their insurer about which provider best suits their needs and preferences [14]. In addition, GPs should not be patients’ only available source of information, since GPs do not routinely discuss referral options adequately with their patients. This is probably because GPs are expected to undertake a long list of tasks [28]. In order to ensure that more enrollees appreciate the healthcare advice services, insurers may need to communicate better about what these services entail and why enrollees could make use of them if they need care.

We studied several differences between groups of enrollees, so that, in the future, insurers could direct their advice to specific groups, or focus on reaching other groups of enrollees. For instance, we found that higher educated enrollees more often indicate that they would probably/certainly not approach their insurer than middle and low educated enrollees. This means that insurers could expect more middle/low educated enrollees on the telephone and could focus on reaching more higher educated enrollees. It could be reasoned that enrollees in bad health may be more aware of insurer’s advice services and are, therefore, more likely to use these services. However, no relationships between respondents' subjective health and their awareness, (potential) use and satisfaction of insurers’ advise services were found. This may be the result of the fact that the number of observations is quite low and future research into these relationships may be useful.

### Strengths and limitations

The strengths of this study are that we used a mixed method design to answer our research questions (website analysis and questionnaire study). Another strength is that the questionnaire was both sent through the internet and by post. However, the respondents were not fully representative of the Dutch population aged 18 years and older as older people were over-represented. We expect this does not affect our results, since all subgroups are of sufficient size and also because we applied a weighting factor. Nevertheless, it can be argued that members of a healthcare panel are more interested in healthcare and therefore might have a more positive attitude towards healthcare advice services. However, we think that this has a minimal influence on our results. The reason for this is that the study concerns specific advice services from the insurer, instead of general healthcare services. A second limitation is that we asked some hypothetical questions in the questionnaire. This could limit the degree to which our findings can be applied in practice because there is a difference between what patients say and what they actually do [29]. An issue relating to this limitation is that the questionnaire could have made enrollees think about the subject of healthcare advice services, while they had not thought about it before. Consequently, our results may overestimate actual figures. For instance, we found that more than one-third of the enrollees who indicated that the potential advice would influence their insurer choice, did not actually know whether their insurer offers healthcare advice. Our questionnaire may have pointed them to the availability of such services, which, in turn, might influence their future choices. Finally, besides respondents’ subjective health, we do not have insight into other variables related to their health or health care use. Consequently, we could not examine the relationship between respondents’ use of healthcare and their awareness, (potential) use and satisfaction of insurers’ advise services.

## Conclusions

Within a system of managed competition, selective contracting and appointing preferred providers are important instruments to enable health insurers to improve their bargaining position in the healthcare purchasing market. Insurers in the Netherlands offer their enrollees extra services, such as healthcare advice, in order to attract enrollees and to ensure that enrollees remain loyal towards their health insurer. This should lead to a better bargaining position vis-à-vis providers on the healthcare purchasing marked, but only if they are able to channel enrollees to preferred providers. We found that, while all insurers offer at least one healthcare advice service, enrollees are generally unaware of these services and only a minority indicate that they ever made use of such a service. Although some people may find being given healthcare advice irrelevant, or they might even not appreciate their insurer’s advice at all, we did find that a reasonable proportion do appreciate their insurers’ healthcare advice services and indicate that they would like to have contact with their insurer if they need care. Therefore, it seems that insurers have so far not made full use of the potential of giving their enrollees healthcare advice. Insurers only seem to reach a limited proportion of the population and they need to think about ways to increase coverage. This study is relevant because it provides insight into a method to attract and channel enrollees about whom we currently know little. Furthermore, it is unique because managed competition had already been introduced into the Netherlands in 2006. Since then insurers have been working hard to fulfil their role as prudent purchasers of care, by developing healthcare services to offer enrollees in order to attract and retain them. Competition in both the healthcare purchasing and health insurance markets should ultimately, according to the policy, contribute to the improvement of healthcare quality and efficiency [30].

## Supporting information

S1 Table The health insurers whose websites we analyzed for the website analysis. (DOCX)

S1 File The questions used for the questionnaire study. (DOCX)
