# Supplementary material for: An assessment of the Dutch experience with health insurers acting as healthcare advisors
Source: PLoS One. 2019 Nov 8;14(11):e0224829. doi: 10.1371/journal.pone.0224829 (PMC6839849; doi:10.1371/journal.pone.0224829)
Supplement: S1 Table — (DOCX) [file pone.0224829.s001.docx]

| **The health insurers whose websites we analyzed for the website analysis** | |
| --- | --- |
| Univé | Salland Zorgverzekeringen |
| Delta Lloyd | Stad Holland Zorgverzekeraar |
| OHRA | ZorgDirect |
| Ditzo | De Friesland |
| Besured | Zorg en Zekerheid |
| IZA | VGZ |
| UMC Zorgverzekering | CZ |
| Zilveren Kruis | IZZ Zorgverzekering (VGZ voor de Zorg: uitvoerder van de IZZ Zorgverzekering) |
| Interpolis | Aevitae |
| OZF | Promovendum |
| Avéro Achmea | National Academic |
| Menzis | De Amersfoortse |
| Anderzorg | HollandZorg |
| ONVZ | Bewuzt |
| FBTO | ZEKUR |
| DSW Zorgverzekeraar | Zorgzaam |
