# Supplementary material for: An assessment of the Dutch experience with health insurers acting as healthcare advisors
Source: PLoS One. 2019 Nov 8;14(11):e0224829. doi: 10.1371/journal.pone.0224829 (PMC6839849; doi:10.1371/journal.pone.0224829)
Supplement: S1 File — (DOCX) [file pone.0224829.s002.docx]

**English translation of the questions**

1. What is your date of birth? *(day - month -year)*

__ __ - __ __ - __ __ __ __

1. Are you a man or a woman?
   - Man
   - Woman
2. Overall, how would you rate your health?
   - Excellent
   - Very good
   - Good
   - Fair
   - Poor

Hereafter, we will ask you several questions about different forms of healthcare advice that your insurer could offer you. For example advice about the most suitable provider, waiting list mediation, assistance with arranging care or assistance with preparing a consultation with a physician.

1. Suppose that you needed one of the healthcare advice services that are mentioned above. Would you contact your health insurer for such a service?

- Certainly not
- Probably not
- Probably
- Certainly
- I don’t know

1. Would you appreciate it if your health insurer would approach you actively with healthcare advice, for instance about the quality of a specific healthcare provider?

- I would strongly disapprove that
- I would disapprove that
- I would approve that
- I would strongly approve that
- I do not have an opinion about this

1. To what degree does the potential that your health insurer could offer healthcare advice influence your choice of health insurer?

- It has no influence
- It has some influence
- It has a major influence
- I don’t know

1. Does your health insurer offer **advice about what the most suitable provider is for you** ? *(multiple answers possible)
   We mean advice about the most suitable provider in your situation.*

- No
- I don’t know
- Yes, I saw that on my health insurer’s website
- Yes, my health insurer informed me about the service by e-mail or by post
- Yes, I saw that in a commercial of my health insurer
- Yes, my health insurer informed me about the service when I asked them a question
- Yes, I found that out in another way, i.e….

1. Does your health insurer offer **waiting list mediation**? *(multiple answers possible)*

*In the event of a long waiting list for treatment with your care provider (for instance a hospital), you can contact your health insurer to apply for waiting time mediation. The insurer will then assess whether an alternative provider is capable of providing appropriate treatment sooner. This is called waiting list mediation.*

- No
- I don’t know
- Yes, I saw that on my health insurer’s website
- Yes, my health insurer informed me about the service by e-mail or by post
- Yes, I saw that in a commercial of my health insurer
- Yes, my health insurer informed me about the service when I asked them a question
- Yes, I found that out in another way, i.e….

1. Does your health insurer offer **assistance with arranging care**? *(multiple answers possible)*

*You can think of, for instance, arranging district nursing or a second opinion.*

- No
- I don’t know
- Yes, I saw that on my health insurer’s website
- Yes, my health insurer informed me about the service by e-mail or by post
- Yes, I saw that in a commercial of my health insurer
- Yes, my health insurer informed me about the service when I asked them a question
- Yes, I found that out in another way, i.e….

1. Does your health insurer offer **assistance with preparing a consultation with a physician**? *(multiple answers possible)*

*You can think of, for instance, assistance with preparing questions you could ask during the consultation.*

- No
- I don’t know
- Yes, I saw that on my health insurer’s website
- Yes, my health insurer informed me about the service by e-mail or by post
- Yes, I saw that in a commercial of my health insurer
- Yes, my health insurer informed me about the service when I asked them a question
- Yes, I found that out in another way, i.e….

1. Several healthcare advice services a health insurer could offer are mentioned below. Would you indicate, per healthcare advice service, if you used it at least once? *(multiple answers possible)*

*Yes, in 2017 Yes, before 2017 No, never used the service*

advice about the most suitable provider ❑ ❑ ❑

waiting list mediation ❑ ❑ ❑

assistance with arranging care ❑ ❑ ❑

assistance with preparing a ❑ ❑ ❑

consultation with a physician

1. Would you indicate, per healthcare advice service, how satisfied you were with the service?

*If you never used the service, you could tick not applicable (na). If you used healthcare advice several times, we ask you to indicate how satisfied you were with those services in general.*

*Very Unsatisfied Not satisfied/ Satisfied Very na, did not*

*unsatisfied not unsatisfied satisfied use*

*the service*

advice about the most ❑ ❑ ❑ ❑ ❑ ❑

suitable provider

waiting list mediation ❑ ❑ ❑ ❑ ❑ ❑

assistance with ❑ ❑ ❑ ❑ ❑ ❑

arranging care

assistance with preparing a ❑ ❑ ❑ ❑ ❑ ❑

consultation with a physician

**Original questions in Dutch**

1. Wat is uw geboortedatum? *(dag - maand - jaar)*

__ __ - __ __ - __ __ __ __

1. Bent u een man of een vrouw?
   - Man
   - Vrouw
2. Hoe zou u over het algemeen uw gezondheid noemen?

❑ Uitstekend
 ❑ Zeer goed
 ❑ Goed
 ❑ Matig
 ❑ Slecht

In dit onderdeel stellen we u een aantal vragen over verschillende van vormen advies die uw zorgverzekeraar u kan bieden. Bijvoorbeeld advies over wat voor u de meest geschikte zorgaanbieder is, wachtlijstbemiddeling, begeleiding bij het regelen van zorg of hulp bij de voorbereiding van een gesprek met een arts.

1. Stel dat u behoefte heeft aan een van de hierboven genoemde vormen van advies. Zou u daar uw zorgverzekeraar voor benaderen?

- Zeker niet
- Waarschijnlijk niet
- Waarschijnlijk wel
- Zeker wel
- Weet ik niet

1. Wat zou u ervan vinden als uw zorgverzekeraar u actief zou benaderen met advies, bijvoorbeeld over de kwaliteit van een specifieke zorgaanbieder?

- Dat zou ik zeer onprettig vinden
- Dat zou ik onprettig vinden
- Dat zou ik prettig vinden
- Dat zou ik zeer prettig vinden
- Daar heb ik geen mening over

1. In hoeverre speelt het advies dat uw zorgverzekeraar kan bieden voor u een rol bij het kiezen van een zorgverzekeraar?

- Dat speelt helemaal geen rol
- Dat speelt een beetje een rol
- Dat speelt een grote rol
- Weet ik niet

1. Kunt u bij uw zorgverzekeraar terecht voor **advies over wat voor u de meest geschikte zorgaanbieder is**? *(meerdere antwoorden mogelijk)*

*We bedoelen hier advies over wat in uw situatie de meest geschikte zorgaanbieder is.*

- Nee
- Weet ik niet
- Ja, dat heb ik gezien op de website van mijn zorgverzekeraar
- Ja, mijn zorgverzekeraar heeft me daarover geïnformeerd per mail of per post
- Ja, dat heb ik gezien in een reclameboodschap van mijn zorgverzekeraar
- Ja, mijn zorgverzekeraar heeft me daarover geïnformeerd toen ik een vraag stelde
- Ja, daar ben ik op een andere manier achter gekomen, namelijk………..

1. Kunt u bij uw zorgverzekeraar terecht voor **wachtlijstbemiddeling**? *(meerdere antwoorden mogelijk)*

*Als het lang duurt voordat u bij uw eigen zorgaanbieder (bijvoorbeeld ziekenhuis) aan de beurt bent, kan de zorgverzekeraar voor u op zoek gaan naar een zorgaanbieder waar u eerder terecht kunt. Dit heet wachtlijstbemiddeling.*

- Nee
- Weet ik niet
- Ja, dat heb ik gezien op de website van mijn zorgverzekeraar
- Ja, mijn zorgverzekeraar heeft me daarover geïnformeerd per mail of per post
- Ja, dat heb ik gezien in een reclameboodschap van mijn zorgverzekeraar
- Ja, mijn zorgverzekeraar heeft me daarover geïnformeerd toen ik een vraag stelde
- Ja, daar ben ik op een andere manier achter gekomen, namelijk………..

1. Kunt u bij uw zorgverzekeraar terecht voor **begeleiding bij het regelen van zorg**?

*(meerdere antwoorden mogelijk)*

*Denk bijvoorbeeld aan het regelen van wijkverpleging of een second opinion.*

- Nee
- Weet ik niet
- Ja, dat heb ik gezien op de website van mijn zorgverzekeraar
- Ja, mijn zorgverzekeraar heeft me daarover geïnformeerd per mail of per post
- Ja, dat heb ik gezien in een reclameboodschap van mijn zorgverzekeraar
- Ja, mijn zorgverzekeraar heeft me daarover geïnformeerd toen ik een vraag stelde
- Ja, daar ben ik op een andere manier achter gekomen, namelijk………..

1. Kunt u bij uw zorgverzekeraar terecht voor **hulp bij de voorbereiding van een gesprek met een arts**? *(meerdere antwoorden mogelijk)*

*Denk bijvoorbeeld aan meedenken over welke vragen u kunt stellen tijdens het gesprek.*

- Nee
- Weet ik niet
- Ja, dat heb ik gezien op de website van mijn zorgverzekeraar
- Ja, mijn zorgverzekeraar heeft me daarover geïnformeerd per mail of per post
- Ja, dat heb ik gezien in een reclameboodschap van mijn zorgverzekeraar
- Ja, mijn zorgverzekeraar heeft me daarover geïnformeerd toen ik een vraag stelde
- Ja, daar ben ik op een andere manier achter gekomen, namelijk………..

1. Hieronder staat een aantal vormen van zorgadvies door een zorgverzekeraar. Wilt u voor de verschillende vormen van advies aangeven of u er wel eens gebruik van heeft gemaakt? *(meerdere antwoorden mogelijk per regel)*

*Ja, in 2017 Ja, vóór 2017 Nee, nooit gebruik*

*van gemaakt*

advies over wat voor u de meest ❑ ❑ ❑

geschikte zorgaanbieder is

wachtlijstbemiddeling ❑ ❑ ❑

begeleiding bij het regelen van zorg ❑ ❑ ❑

hulp bij de voorbereiding van ❑ ❑ ❑

een gesprek met de arts

1. Wilt u hieronder voor de verschillende soorten advies aangeven hoe tevreden u daarover was?

*Als u nooit gebruik heeft gemaakt van het advies kunt u niet van toepassing (n.v.t.) aankruisen. Als u meerdere keren gebruik heeft gemaakt van het advies vragen we u aan te geven hoe tevreden u in het algemeen was met de adviezen.*

*Heel Ontevreden Niet tevreden/ Tevreden Heel n.v.t., geen*

*ontevreden niet ontevreden tevreden gebruik van*

*gemaakt*

advies over wat voor u ❑ ❑ ❑ ❑ ❑ ❑

de meest geschikte

zorgaanbieder is

wachtlijstbemiddeling ❑ ❑ ❑ ❑ ❑ ❑

begeleiding bij het ❑ ❑ ❑ ❑ ❑ ❑

regelen van zorg

hulp bij de voorbereiding ❑ ❑ ❑ ❑ ❑ ❑

van een gesprek met de arts
